# Independent Validation of Sepsis Index for Sepsis Screening in the Emergency Department

**DOI:** 10.3390/diagnostics11071292

**Published:** 2021-07-19

**Authors:** Luisa Agnello, Alessandro Iacona, Salvatore Maestri, Bruna Lo Sasso, Rosaria Vincenza Giglio, Silvia Mancuso, Anna Maria Ciaccio, Matteo Vidali, Marcello Ciaccio

**Affiliations:** 1 Department of Biomedicine, Neurosciences and Advanced Diagnostics, Institute of Clinical Biochemistry, Clinical Molecular Medicine and Laboratory Medicine, University of Palermo, 90127 Palermo, Italy; luisa.agnello@unipa.it (L.A.); bruna.losasso@unipa.it (B.L.S.); rosaria.vincenza.giglio@alice.it (R.V.G.); silvia.mancuso.biologo@gmail.com (S.M.); 2 Department of Laboratory Medicine, University Hospital “P. Giaccone”, 90127 Palermo, Italy; alessandro.iacona84@gmail.com (A.I.); maestrisalvatore@gmail.com (S.M.); 3 Unit of Clinical Biochemistry, University of Palermo, 90127 Palermo, Italy; amciaccio21@gmail.com; 4 Foundation IRCCS Ca’ Granda Ospedale Maggiore Policlinico, 20122 Milan, Italy; matteo.vidali@gmail.com

**Keywords:** sepsis, biomarker, screening, CBC, monocytes, MDW, MMV

## Abstract

(1) Background: The early detection of sepsis is still challenging, and there is an urgent need for biomarkers that could identify patients at a high risk of developing it. We recently developed an index, namely the Sepsis Index (SI), based on the combination of two CBC parameters: monocyte distribution width (MDW) and mean monocyte volume (MMV). In this study, we sought to independently validate the performance of SI as a tool for the early detection of patients at a high risk of sepsis in the Emergency Department (ED). (2) Methods: We enrolled all consecutive patients attending the ED with a request of the CBC. MDW and MMV were measured on samples collected in K3-EDTA tubes on the UniCel DxH 900 haematology analyser. SI was calculated based on the MDW and MMV. (3) Results: We enrolled a total of 703 patients stratified into four subgroups according to the Sepsis-2 criteria: control (498), infection (105), SIRS (52) and sepsis (48). The sepsis subgroup displayed the highest MDW (median 27.5, IQR 24.6–32.9) and SI (median 1.15, IQR 1.05–1.29) values. The ROC curve analysis for the prediction of sepsis showed a good and comparable diagnostic accuracy of the MDW and SI. However, the SI displayed an increased specificity, positive predictive value and positive likelihood ratio in comparison to MDW alone. (4) Conclusions: SI improves the diagnostic accuracy of MDW for sepsis screening.

## 1. Introduction

Sepsis is a highly complex disease caused by a deregulated host response to infection. It has been recently recognised as a global health priority by the World Health Organization due to its high mortality and morbidity [1]. Accordingly, the rapid detection of sepsis is crucial in order to prevent adverse outcomes and reduce mortality by promptly starting the treatment. Indeed, it has been estimated that each hour of treatment delay is associated with a 7–10% increase in sepsis-related mortality [2]. However, the early diagnosis of sepsis remains challenging, because it is characterised by no specific signs and symptoms. Thus, many efforts have been made to identify reliable biomarkers for screening patients at a high risk of sepsis [3,4,5,6,7]. Among all the biomarkers investigated, the parameters belonging to the Complete Blood Count (CBC) have emerged as precious tools providing a wealth of information on individual health statuses. In addition to the traditional CBC parameters, the new generation of haematological analysers provide several parameters, called cell population data (CPD), which reflect the morphological and functional characteristics of leukocytes [8]. The monocyte distribution width (MDW) is a CPD parameter automatically generated by last-generation DxH 900 haematology analysers (Beckman Coulter, Inc., Brea, CA, USA). The Food and Drug Administration approved the MDW, also known as the Early Sepsis Indicator (ESId), as a biomarker for the identification of patients with sepsis or at risk of developing sepsis in the Emergency Department (ED). Specifically, it represents a morphometric indicator of monocyte size variability similar to the red blood cell distribution width. Several authors have assessed the diagnostic performance of MDW as a biomarker of sepsis in different acute clinical settings, including the ED and intensive care unit (ICU), achieving promising results [9,10,11]. We also found that the MDW had high sensitivity and specificity for sepsis screening both in the ED and in ICU [12,13,14]. Additionally, we established the reference intervals of the MDW in a healthy population of blood donors [15]. Although, in our studies, the MDW showed a good diagnostic performance for sepsis screening, we observed that some individuals were not correctly classified according to its value, especially in the ED. Accordingly, we developed an index, namely the Sepsis Index (SI), to improve the specificity of the MDW [16]. SI is based on the combination of MDW with another CBC parameter related to the morphological characteristics of monocytes, the mean monocyte volume (MMV).

The aim of this study was to independently validate the SI for early identifying patients at a high risk of sepsis in ED.

## 2. Materials and Methods

### 2.1. Study Population

This is a retrospective, observational, single-centre cohort study performed at the University Hospital “P. Giaccone” of Palermo, Italy. The study was approved by the Institutional Review Board of the University Hospital of Palermo (nr 07/2019) on 10 July 2019. 

Eligible patients were all consecutive adult patients (≥18 years of age) admitted to the ED from October to November 2020, with a request of a complete CBC as part of the initial evaluation.

Exclusion criteria were: (i) age < 18 years;,(ii) incomplete data collection, (iii) discharge from the ED within 2 h from ED presentation, (iv) readmission to the ED within 12 h; (v) inadequate blood samples (e.g., analysed >4 h after collection); (vi) failure to determine the MDW parameter and (vii) the presence of clinical conditions, such as haematological disorders (*n* = 11) or chronic treatment with an immunosuppressant (*n* = 6), which could influence the MDW values.

For each patient, we recorded demographic, clinical and laboratory data. Based on the Sepsis-2 criteria [17], the patients were classified into 4 subgroups: controls (patients without infection, Systemic Inflammatory Response Syndrome (SIRS) and sepsis); SIRS (at least two SIRS criteria); infection (patients without sepsis and with zero or one SIRS criterion) and sepsis (patients with a confirmed or suspected infection and SIRS). The SIRS criteria were tachycardia, defined as heart rate >90 beats/min; tachypnoea, defined as respiratory rate >20 breaths/min; fever or hypothermia, defined as temperature >38 or <36 °C, respectively, and leucocytosis and leukopenia, defined as white blood cells (WBC) >12 × 10^9^/L and <4 × 10^9^/L, respectively. The infection was defined according to the clinical, imaging and laboratory test findings. The classification of the patients was performed by a retrospective review of the electronic medical records by four investigators independently.

### 2.2. MDW and Sepsis Index Evaluation

The MDW was measured on whole-blood venous samples collected in K3-EDTA tubes on the UniCel DxH 900 haematology analyser (Beckman Coulter, Inc., Brea, CA, USA) within 2 h from the collection, as recommended by the manufacturer, after the laboratory analysis ordered by the ED clinicians were completed. The SI was calculated for each patient by applying the formula based on the values of the MDW and MMV, as previously described [16].

### 2.3. Statistical Analysis

Statistical analyses were performed by SPSS statistical software v.17.0 (SPSS Inc., Chicago, IL, USA) and R Language v.4.0.3 (R Foundation for Statistical Computing, Vienna, Austria). Normality distribution was assessed preliminarily by q-q plot and Shapiro–Wilk tests. Quantitative variables were expressed as the median and interquartile range (IQR), while qualitative variables as the absolute and relative frequencies. Differences between groups for continuous variables were estimated by the nonparametric Kruskal–Wallis test (if >2 groups) or Mann–Whitney *U* test (with Bonferroni’s correction when needed). Diagnostic accuracy for the prediction of sepsis was evaluated by a Receiver Operating Characteristic (ROC) curve analysis and reported as the Area Under the Curve (AUC) and 95% confidence interval. Differences between the AUCs were evaluated by the DeLong method.

## 3. Results

Seven hundred and three patients (Controls, *n* = 498, Infection, *n* = 105, SIRS, *n* = 52 and Sepsis, *n* = 48) were evaluated. The levels of the haematological parameters, including the white blood cells (WBC), monocytes (MO), MMV, the standard deviation monocyte volume (SDMV), MDW and SI, are shown in Table 1. Taking into account Bonferroni’s correction, the patients with sepsis displayed significantly higher levels of WBC (*p* < 0.001 vs. the controls and infection); MMV (*p* < 0.001 vs. all groups) and SDMV (*p* < 0.001 vs. all groups) but not MO (*p* = 0.588 vs. controls, *p* = 1.000 vs. infection and *p* = 0.732 vs. SIRS) (Table 1).

**Figure 1:**
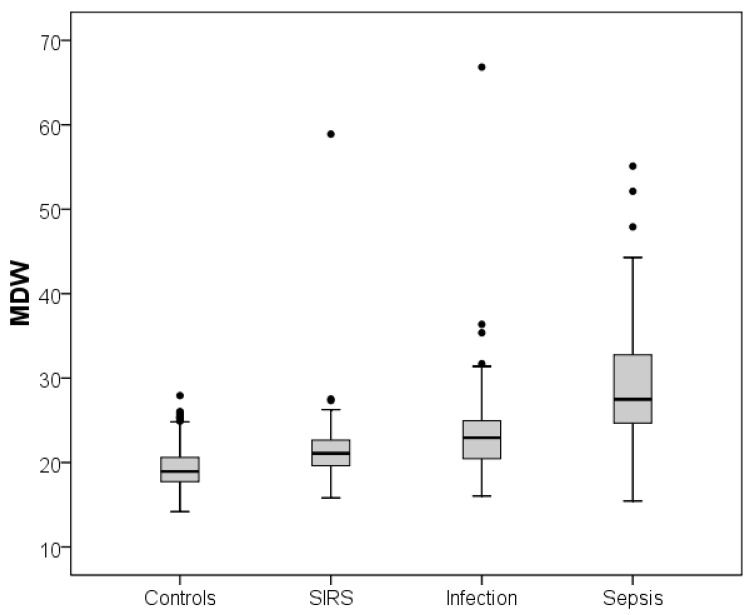
Distribution of the MDW values in the subgroups investigated. In the boxplot (or box-and-whisker plot), the central box represents the values from the lower to upper quartiles (25th to 75th percentiles) or interquartile range (IQR). The middle line represents the median. Whiskers sprout from the two ends of the box up to the observations within, respectively, the lower quartile minus 1.5 times the IQR or the upper quartile plus 1.5 times the IQR. Larger observations are displayed as points and represent outliers.

**Figure 2:**
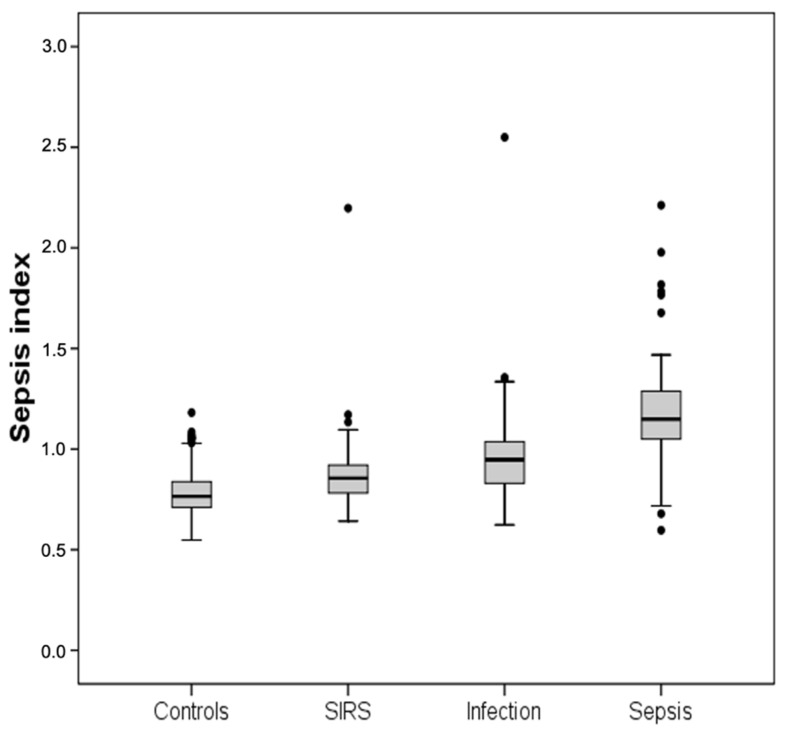
Distribution of the Sepsis Index values in the subgroups investigated. In the boxplot (or box-and-whisker plot), the central box represents the values from the lower to upper quartiles (25th to 75th percentiles) or interquartile range (IQR). The middle line represents the median. Whiskers sprout from the two ends of the box up to the observations within, respectively, the lower quartile minus 1.5 times the IQR or the upper quartile plus 1.5 times the IQR. Larger observations are displayed as points and represent outliers.

The sepsis subgroup displayed the highest MDW (median 27.5, IQR 24.6–32.9) and SI (median 1.15, IQR 1.05–1.29) values. In particular, the MDW and SI were significantly higher in the sepsis subgroup than the other three subgroups (*p* < 0.001 for all comparisons, taking into account Bonferroni’s correction) (Table 1 and Figure 1 and Figure 2).

**Figure 3:**
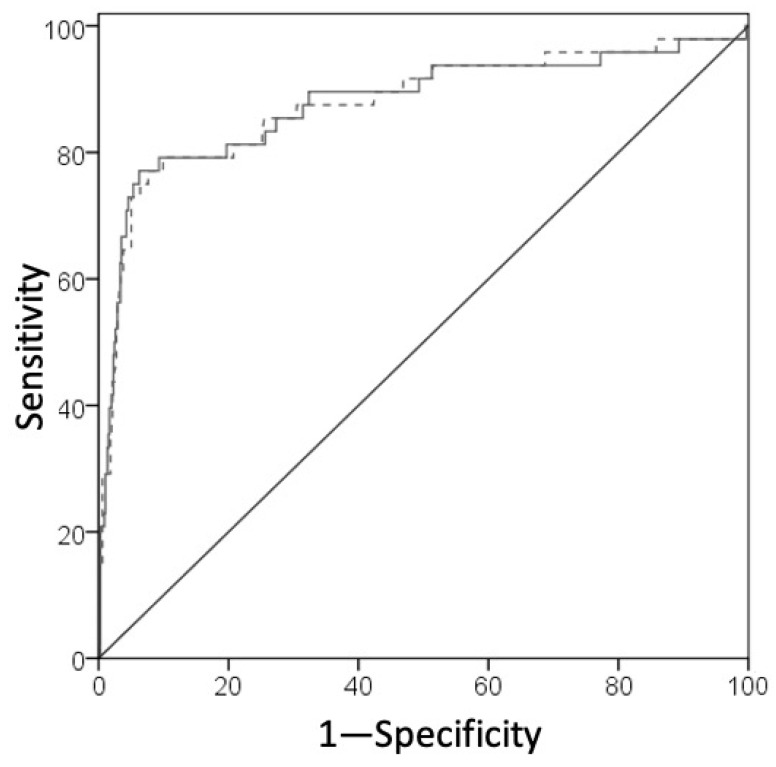
ROC curve analysis for comparison of the MDW (dashed) and Sepsis Index (solid) levels in sepsis prediction. The area under the curve (AUC) represents the test accuracy. The diagonal line, with an AUC of 0.5, indicates a perfect chance.

At the ROC curve analysis for the prediction of sepsis, the AUCs of MDW and SI were, respectively, 0.876 (95% CI 0.812–0.942) and 0.877 (95% CI 0.810–0.944) (Figure 3). According to DeLong’s test, no significant difference was observed between the AUCs of the MDW and SI (*p* = 0.889).

Using the previously established decisional cut-off values of 23 and 1 for the MDW and SI, respectively [13,16], we found out that, in this validation cohort, the sensitivity, specificity, positive predictive value (PPV), negative predictive value (NPV), positive likelihood ratio (LR+) and negative likelihood ratio (LR-) for sepsis were, respectively, 0.79 (MDW) vs. 0.79 (SI), 0.84 vs. 0.91, 0.27 vs. 0.38, 0.98 vs. 0.98, 5.08 vs. 8.52 and 0.25 vs. 0.23.

## 4. Discussion

In this study, we validated the performance of the Sepsis Index for the screening of patients at a high risk of developing sepsis in the ED. We previously developed the SI by using the data from a large cohort of consecutive patients admitted to the ED. The SI is based on the relationship between two CBC parameters: MDW and MMV, which provide complementary information on the monocytes’ morphological features. Thus, the SI allows capturing the morphological variability of the monocyte population during the early stages of sepsis. Indeed, monocytes represent the first line of defence against invading pathogens [18]. The activation of monocytes induces functional and morphological changes leading to a highly heterogeneous population, especially during the early phases of sepsis. The SI, by combining the MDW and MMV, detects such heterogeneity.

The findings of this validation study confirmed that the SI improves the diagnostic performance of the MDW by ameliorating its specificity without compromising its sensitivity and by reducing the rate of false positives. Thus, the SI represents a reliable tool for sepsis screening in ED. Our findings encourage the implementation of the SI in the haemocytometers in order to make it automatically available to all clinicians together with the basic CBC parameters.

To date, hundreds of sepsis biomarkers have been described in the literature [19]. However, most of them present some limitations, such as a poor diagnostic performance, elevated cost, additional tests to order and a long turn-around time (TAT). The use of the SI, which is based on two CBC parameters, represents an appealing tool for improving the clinical outcome in patients with sepsis. Indeed, a CBC has several advantages: (i) it represents the first-line laboratory test most commonly ordered in all clinical settings, from the ED to ICU; (ii) clinicians routinely request a CBC as part of the management of patients and (iii) it is easy to perform, cheap and has a fast TAT.

It is important to emphasize that the SI is not a diagnostic biomarker of sepsis, but it identifies patients at a high risk of developing it. Thus, a value of the SI above the decisional cut-off should be interpreted as an alert and should induce clinicians to investigate the possible presence of sepsis. Notable, sepsis is a complex disease, and the diagnosis relies on the integration of clinical and laboratory findings.

In conclusion, we developed and validated a tool for sepsis screening in ED. The SI may assist clinicians in identifying patients at a risk of developing sepsis also when such a condition is not suspected. Indeed, the SI could be potentially always available to clinicians together with the routine CBC parameters. The strength of our study is that it was performed in a “real world” setting, including patients admitted to the ED as controls. Several studies use healthy individuals non-hospitalised individuals as controls, leading to a misleading evaluation of the real performance of a biomarker, as stated by Heffernan and Denny [20].

Noteworthy, the SI has been developed and tested in a cohort of patients from ED and by applying Sepsis-2 criteria. Thus, its use is validated in this clinical setting. Before introducing it in clinical practice, its performance should be tested in other clinical wards.

## Figures and Tables

**Table 1:** Demographic and biochemical characteristics of the subgroups investigated.

DATA (Median, IQR), *n* (%)	Control (*n* = 498)	SIRS (*n* = 52)	Infection (*n* = 105)	Sepsis (*n* = 48)	*p*
Demographic					
Age, years	59 (45–74)	64 (45–79)	57 (43–71)	65 (45–75)	0.323
Sex, M (%)	40%	52%	61%	48%	
Biochemical					
WBC, 10^9^/L	8.95 (7.23–11.50)	13.61 (12.28–15.87)	9.63 (6.96–11.58)	14.77 (7.52–20.86)	**<0.001**
MO, 10^9^/L	0.66 (0.53–0.86)	0.97 (0.70–1.35)	0.76 (0.52–1.02)	0.79 (0.48–1.31)	**<0.001**
MMV	174 (169–178)	174 (169–181)	181 (175–187)	192 (182–201)	**<0.001**
SDMV	19.1 (18.0–20.7)	20.4 (19.1–22.5)	22.2 (19.8–24.1)	25.1 (22.9–27.8)	**<0.001**
MDW	18.9 (17.7–20.6)	21.1 (19.6–22.7)	22.9 (20.5–25.1)	27.5 (24.6–32.9)	**<0.001**
Sepsis Index	0.77 (0.71–0.84)	0.86 (0.78–0.92)	0.95 (0.83–1.05)	1.15 (1.05–1.29)	**<0.001**

IQR, interquartile range; WBC, white blood count; MO, monocytes; MMV, mean monocytes volume; SDMV, standard deviation monocytes volume and MDW, monocyte distribution width. The *p*-value represents the statistical significance obtained with an overall or omnibus test (Kruskal–Wallis). Post hoc analyses with the Mann–Whitney test for all 6 possible comparisons were conducted after a statistically significant omnibus test (see text). Bold values denote statistical significance at the *p* < 0.05 level.

## Data Availability

Derived data supporting the findings of this study are available from the corresponding author on request.
